# Sexual Shape Variation and Allometric Effects in Guinea Pig (*Cavia porcellus*) Skulls

**DOI:** 10.3390/ani15233453

**Published:** 2025-11-30

**Authors:** Ebru Eravci Yalin, Tomasz Szara, Ebuderda Günay, Ana Pešić, Nicoleta Manuta, Barış Can Güzel, Muhammed Taha Temir, Ozan Gündemir

**Affiliations:** 1 Department of Surgery, Faculty of Veterinary Medicine, Istanbul University-Cerrahpasa, Istanbul 34320, Türkiye; ebru.eravciyalin@iuc.edu.tr; 2 Department of Morphological Sciences, Institute of Veterinary Medicine, Warsaw University of Life Sciences-SGGW, 02-776 Warsaw, Poland; 3 Department of Wild Animal Disease and Ecology, Faculty of Veterinary Medicine, Istanbul University-Cerrahpasa, Istanbul 34320, Türkiye; ebuderda.gunay@iuc.edu.tr; 4 Department of Equine, Small Animal, Poultry and Wild Animal Diseases, Faculty of Veterinary Medicine, University of Belgrade, 11000 Belgrade, Serbia; ana.pesic@vet.bg.ac.rs; 5 Institute of Graduate Studies, Istanbul University-Cerrahpasa, Istanbul 34320, Türkiye; nicoletamanuta@ogr.iuc.edu.tr; 6 Department of Anatomy, Faculty of Veterinary Medicine, Siirt University, Siirt 56100, Türkiye; baris.guzel@siirt.edu.tr; 7 Department of Veterinary Radiology, Faculty of Veterinary Medicine, Istanbul University-Cerrahpasa, Istanbul 34320, Türkiye; tahatemir@iuc.edu.tr; 8 Department of Anatomy, Faculty of Veterinary Medicine, Istanbul University-Cerrahpasa, Istanbul 34320, Türkiye; 9 Osteoarchaeology Practice and Research Centre, Istanbul University-Cerrahpasa, Istanbul 34320, Türkiye

**Keywords:** 3D imaging, allometry, cranial morphology, computed tomography, guinea pig, sexual dimorphism

## Abstract

The skull provides essential diagnostic features for species identification and sex determination. Cranial sexual dimorphism in guinea pigs (*Cavia porcellus*) is particularly important to understand, as these animals are frequently used in experimental research and veterinary practice, yet detailed morphometric evaluations remain limited. This study aimed to assess cranial size and shape variation between sexes using three-dimensional geometric morphometric (GM) methods. Computed tomography scans of 30 clinically healthy guinea pigs were used to reconstruct three-dimensional skull models. Twenty-one anatomical landmarks were digitized, and the dataset was subjected to generalized Procrustes analysis, followed by principal component analysis, Procrustes ANOVA, and regression-based allometric assessments. The results revealed pronounced sexual dimorphism. Males exhibited significantly larger centroid sizes and more robust cranial morphologies, whereas females presented smaller and more gracile skull forms. Procrustes ANOVA confirmed significant shape differences between sexes (*p* < 0.01), with size being the primary driver of morphological divergence. Body weight had a weaker but detectable influence on cranial variation, although its effect diminished after accounting for centroid size (R^2^: 0.085). These findings demonstrate that three-dimensional geometric morphometrics can effectively detect subtle patterns of cranial dimorphism in guinea pigs. Beyond their anatomical relevance, the results provide a valuable reference for veterinary sciences, taxonomy, and future biomedical research requiring precise morphological characterization. These findings show that three-dimensional geometric morphometrics can effectively detect subtle patterns of sexual dimorphism in cranial shape, which is particularly relevant for sex identification in skeletal collections and for the development of comparative anatomical databases in veterinary and experimental research.

## Simple Summary

This study analyzed sexual dimorphism and allometric effects in the skulls of guinea pigs (*Cavia porcellus*) using three-dimensional (3D) geometric morphometrics. Thirty adult specimens were scanned by computed tomography, and 21 cranial landmarks were digitized for statistical analysis. Males exhibited significantly larger and more robust skulls than females, which displayed smaller and more gracile cranial morphology. Procrustes-based allometric regression analysis indicated that shape variation was predominantly size-dependent, with body weight exerting negligible influence on cranial shape. These results provide valuable reference data for veterinary diagnostics and for future comparative studies on cranial morphology in guinea pigs and other small mammals.

## 1. Introduction

Guinea pigs (*Cavia porcellus*), small hystricomorph rodents domesticated from South American wild cavies, are widely used as biomedical models [1]. Their robust skull, characteristic of herbivorous rodents, features a toothless diastema and large auditory bullae [2]. Domestication has had a minimal effect on cranial morphology; domestic skull length is ~5% shorter than that of its wild ancestors [3]. Unlike many rodents (e.g., laboratory rats) with marked cranial size dimorphism [4], guinea pigs show minimal sexual dimorphism in skull size. Although some mandibular measurements differ between sexes, overall cranial size is similar in males and females [5]. Skull growth is rapid postnatally, with more than 80% of head dimension increase occurring within the first eight weeks [6]. This precocial development underscores the importance of examining cranial morphology across ontogenetic stages and sexes.

Computed tomography (CT) imaging and three-dimensional (3D) modeling have revolutionized the anatomical analysis of skull morphology. CT provides detailed cross-sectional views of internal structures, yielding greater anatomical detail and contrast than traditional planar radiographs [7,8,9]. This is particularly advantageous for complex regions such as the craniofacial skeleton. CT scans enable clear visualization of components, including the nasal cavity, paranasal sinuses, bullae, and braincase. These structures are often superimposed or obscured in standard radiographs. Accordingly, CT has become a standard tool in veterinary anatomy and diagnostic imaging for small animals [8]. Beyond two-dimensional slices, modern imaging software enables 3D reconstruction of CT data into virtual bone models [10]. These 3D models can be rotated, sectioned, and measured. They serve as valuable anatomical references for research and clinical applications. For instance, volume-rendered 3D skull models have been used to identify and label fine osteological features in domestic animals [10]. They have also been used to create physical replicas via rapid prototyping (3D printing) for educational or surgical planning purposes [11]. The integration of CT with digital modeling also permits precise, non-destructive quantification of cranial metrics (e.g., distances, angles, volumes, surface areas) [5]. Recent studies have applied these techniques to assess skull development and variation in various species. For example, high-resolution CT analyses have mapped bone architecture and changes in bone thickness in growing guinea pig skeletons [12]. Similarly, CT-based 3D morphometric evaluations have characterized age-related cranial changes in other small mammals [13]. These advances in imaging and modeling provide a foundation for comprehensive geometric analyses.

Geometric morphometrics (GM) provides a powerful framework for quantifying and analyzing biological shape variation [14,15]. Unlike traditional morphometric approaches that rely on linear measurements or ratios, GM uses the coordinates of anatomical landmarks to capture the geometry of structures in two or three dimensions [16,17]. By statistically superimposing landmark configurations (e.g., via Procrustes alignment), differences in shape can be isolated from differences in size, allowing researchers to examine subtle morphological variations and allometric patterns [18]. The method also enables the visualization of shape differences through deformation grids or reconstructed outlines, often utilizing techniques such as thin-plate spline interpolation [19,20]. Pioneering works laid the groundwork for this approach in the early 1990s [16,17], and subsequent developments have made GM widely accessible and applicable across various disciplines [21]. Today, GM is employed extensively in evolutionary biology, paleontology, and veterinary anatomy to compare forms and study developmental or evolutionary changes in shape [20,21,22]. For instance, it has been used to quantify skull shape variation among species, to investigate modularity and integration of cranial structures, and to discern the effects of domestication on morphology [3]. Importantly, GM is also sensitive enough to detect morphological differences linked to sex or other biological factors that might not be evident from raw measurements alone [18]. Recent studies have demonstrated the utility of GM in distinguishing male vs. female individuals based on cranial shape in various animals [23]. Notably, GM approaches can be integrated with other techniques as well, for example, [24] combined landmark-based shape data with finite element modeling to examine how skull shape variation affects biomechanical performance in rodents.

Despite the guinea pig’s importance as a laboratory animal, its cranial morphology has not yet been thoroughly investigated using the modern approaches described above. Guinea pigs have been the subject of conventional morphometric studies. However, no study to date has applied landmark-based geometric morphometrics to capture the overall shape of the guinea pig cranium. This represents a significant gap, given that GM has proven effective for elucidating cranial shape dimorphism in other animals [25,26]. This study provides CT-based three-dimensional geometric morphometric data on cranial sexual dimorphism in guinea pigs, thereby contributing new reference information to the veterinary and experimental cranial morphology literature. To address this gap, the present study aims to integrate CT-based 3D modeling with geometric morphometric analysis to provide a comprehensive evaluation of guinea pig skull shape. Using high-resolution CT scans, we reconstructed accurate 3D models of guinea pig crania, on which a set of homologous landmarks was digitized for GM analysis. By comparing these landmark configurations, we aim to characterize the normal range of skull shape in this species and to investigate any systematic differences related to sex or age. In particular, we investigate whether subtle shape dimorphisms exist between male and female guinea pigs that have gone undetected by traditional measurements. The overarching goal is to enrich the anatomical understanding of the guinea pig skull through a state-of-the-art 3D morphometric approach, establishing a baseline for this species and demonstrating the value of combining imaging and GM techniques in veterinary morphology.

## 2. Materials and Methods

### 2.1. Samples

The study was conducted on 30 clinically healthy adult guinea pigs (*Cavia porcellus*) from a single breeding line maintained at the Istanbul Zoo (Park of Istanbul). Sex was determined from the Park animal records, in which the sex of each guinea pig was documented prior to CT scanning. All animals were housed under comparable husbandry conditions and were older than one year, i.e., skeletally mature adults as judged from body size, dentition, and general physiological appearance. Body weight was available for 28 of the 30 individuals included in the study, ranging from 550 to 880 g in males (mean ± SD, 741.1 ± 96.6 g) and from 410 to 710 g in females (mean ± SD, 597.9 ± 76.8 g). Individuals were presented to the Istanbul University–Cerrahpaşa, Faculty of Veterinary Medicine, Research and Application Animal Hospital for routine health monitoring and therefore represent a convenience clinical sample rather than a randomly selected population. Animals showing obvious cranial asymmetries or dental malocclusion on clinical records or CT images were excluded from the study. All animals were clinically examined prior to imaging, and only individuals without systemic disorders, traumatic histories, or cranial deformities were included in the study.

Cranial imaging was performed using a 128-slice Siemens Definition AS Plus CT scanner. The acquisition protocol was standardized across all animals to ensure comparability of cranial measurements. Scans were obtained with a slice thickness of 0.6 mm, 120 kVp tube voltage, and 240 mAs tube current, providing high-resolution volumetric data suitable for morphometric evaluation. Animals were positioned in sternal recumbency with the head aligned in the mid-sagittal plane to minimize positional artifacts and maintain reproducibility across individuals.

All acquired images were reviewed by board-certified veterinary radiologists, who confirmed the absence of any congenital malformations, fractures, neoplastic lesions, or other pathological conditions that might interfere with cranial morphology. Only data from healthy animals were retained for subsequent morphometric analyses.

### 2.2. D Reconstruction and Landmark Data Acquisition

The cranial CT datasets obtained from each individual were exported in Digital Imaging and Communications in Medicine (DICOM) format and imported into 3D Slicer (version 5.10.0) with the SlicerMorph extension for processing and landmarking. Segmentation and three-dimensional reconstruction of the skulls were performed using 3D Slicer software. The bone was segmented using intensity-based thresholding combined with region growing. Because Hounsfield Unit (HU) values for cranial bone varied slightly among individuals and scan settings, a single fixed HU range was not applied. Instead, for each dataset, the lower and upper thresholds were adjusted interactively around the bone peak in the intensity histogram, while simultaneously inspecting axial, sagittal, and coronal slices to ensure the inclusion of all osseous tissue and the exclusion of adjacent soft tissues and artifacts.

The segmented volumes were subsequently rendered into three-dimensional polygonal surface models and exported in Polygon File Format (PLY) for geometric morphometric processing. A very mild level of mesh processing was applied, utilizing Laplacian surface smoothing and low-intensity mesh decimation to reduce noise and file size while preserving anatomical fidelity. [5,7,27].

**Figure 1:**
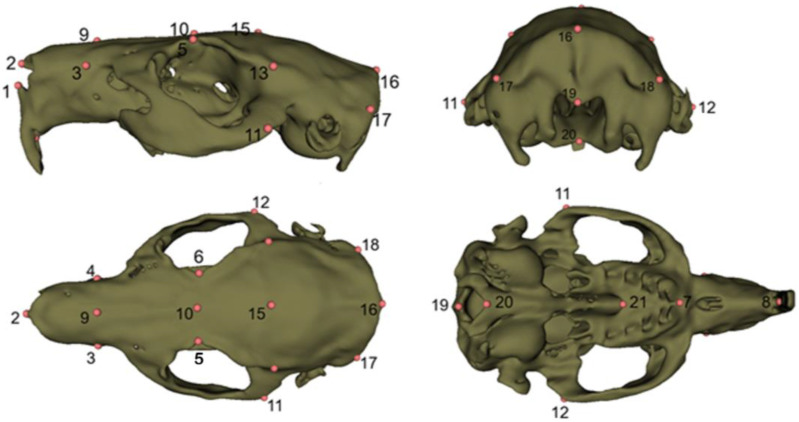
Landmarks.

For morphometric analysis, a predefined set of 3D anatomical landmarks was placed on each skull model using the Markups (fiducial) tools within the SlicerMorph workflow in 3D Slicer, ensuring consistency across all specimens [27,28]. Landmarks were chosen based on reproducibility, visibility in CT-derived 3D models, and relevance to cranial morphology [29] (Figure 1, Table 1). Intra-observer repeatability was checked by relandmarking a subset of specimens, and Procrustes error indicated high repeatability of landmark placement. Landmark coordinates were then exported as FCSV files for subsequent geometric morphometric analyses (Table S1). All digitization was performed by the same observer to minimize inter-observer error.

### 2.3. Statistical Analyses

All statistical analyses were performed in R version 4.4.0 (R Core Team, Vienna, Austria) using the geomorph package (v. 4.0.9) for geometric morphometric analyses. Landmark configurations were subjected to Generalized Procrustes Analysis (GPA) in order to remove effects of translation, rotation, and scale, and to obtain shape variables (Procrustes coordinates) and centroid size as a proxy for overall skull size [30]. To evaluate sexual dimorphism in size, centroid size was compared between males and females using an ANOVA test. Mean values were calculated for each sex, and group differences were quantified.

Differences in centroid size between sexes were assessed using Welch’s two-sample *t*-test. Prior to the analysis, univariate distributional assumptions for centroid size were examined by inspecting histograms and Q–Q plots, as well as by applying Shapiro–Wilk tests separately within each sex. Homogeneity of variances was evaluated using Levene’s test. As a measure of effect size, we computed Hedges’ g (small-sample corrected Cohen’s d) with 95% confidence intervals. The distribution of centroid size by sex was visualised using violin/box plots overlaid with individual data points.

To test for shape differences between sexes, a Procrustes ANOVA (procD.lm) was conducted with sex as a grouping factor. Effect sizes, F-statistics, and permutation-based significance tests (999 iterations) were used to assess the contribution of sex to shape variation. Mean shape configurations for males and females were calculated, and the Procrustes distance between them was computed to quantify morphological divergence.

Patterns of shape variation were explored using Principal Component Analysis (PCA) on the Procrustes coordinates. The first three PCs were examined, and convex hulls were drawn to visualize the distribution of males and females in morphospace. Morphological interpretations were made by comparing shape changes along PC1–PC3. Shape changes were visualized on three-dimensional skull models by warping them to represent the negative and positive extremes along the principal component axes.

Sexual classification based on cranial shape was evaluated using linear discriminant analysis (LDA) on principal component (PC) scores derived from the Procrustes-aligned landmark configurations. To reduce dimensionality while avoiding circular feature selection, we retained the first three PCs; this choice was made a priori based on variance explained and was not tuned to maximise classification accuracy. LDA was then performed using these three PCs as predictors and sex as the grouping factor.

Classification performance was assessed using leave-one-out cross-validation (LOOCV). We report the overall accuracy, as well as the cross-validated sensitivity and specificity, for each sex. To evaluate whether the observed accuracy was greater than expected by chance, we implemented a permutation-based LOOCV procedure in which group labels were randomly permuted 999 times and, for each permuted dataset, the entire LDA + LOOCV pipeline was re-run to obtain an empirical null distribution of accuracies. A permutation *p*-value was calculated as the proportion of permuted accuracies greater than or equal to the observed accuracy. Ninety-five percent confidence intervals for the observed accuracy were obtained using exact binomial (Clopper–Pearson) intervals.

The effects of allometry were tested by regressing shape on centroid size and body weight separately, using Procrustes regression (procD.lm). Additionally, a combined model incorporating both centroid size and body weight was fitted to assess their relative contributions. For each model, permutation tests (999 iterations) were used to evaluate significance. Body weight was available for all individuals included in the analyses. All models, including body weight as a covariate, were therefore fitted using only observed body weight values, without any group-mean imputation of missing data.

Cranial shape was analyzed using Procrustes ANOVA/regression in the R package geomorph (v.4.0.9). To evaluate sexual allometry, we fitted a single multivariate model in which Procrustes shape coordinates were regressed on log-transformed centroid size and sex, including their interaction: shape ~ log(centroid size) + sex + log(centroid size):sex.

Where multiple related hypotheses were tested (e.g., comparisons of PC scores between sexes), *p*-values were adjusted for multiple comparisons using the Benjamini–Hochberg procedure to control the false discovery rate (FDR). All results were reported as effect sizes (R^2^), F-statistics, Z-scores, and permutation-based *p*-values, with significance accepted at *p* < 0.05.

## 3. Results

### 3.1. Size and Shape

**Figure 2:**
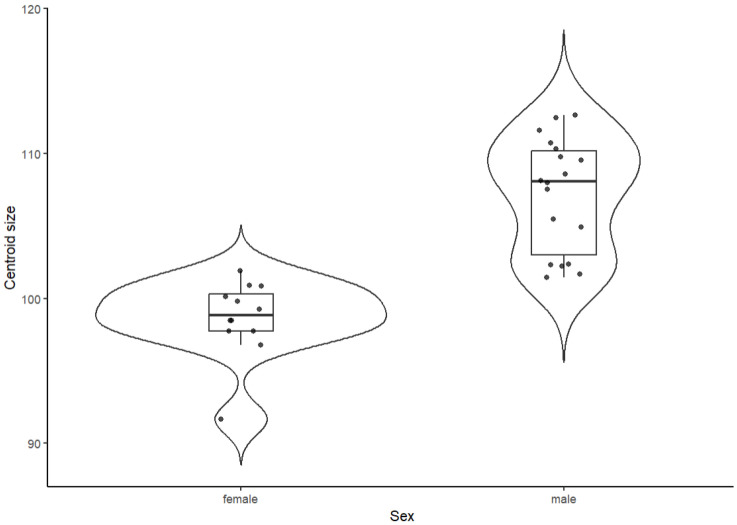
Sex-related differences in centroid size in guinea pigs.

Centroid size differed strongly between sexes. Female guinea pigs had smaller skulls (mean ± SD: 98.6 ± 2.7) than males (107.2 ± 3.9). Welch’s *t*-test indicated a highly significant difference in centroid size between sexes (t = −7.14, df = 27.95, *p* = 9.1 × 10^−8^), with the 95% confidence interval for the mean difference (female–male) ranging from −11.03 to −6.11. The magnitude of this difference was very large, as reflected by a Hedges’ g of 2.40 (males larger; 95% CI: 1.44–3.37). Shapiro–Wilk tests suggested no strong deviations from normality within each sex (female: *p* = 0.056; male: *p* = 0.079), and Levene’s test provided only weak evidence for variance heterogeneity (F (1,28) = 3.07, *p* = 0.091), supporting the use of Welch’s unequal-variance *t*-test. The sex-specific distributions of centroid size, including individual data points, are shown in Figure 2.

Procrustes ANOVA revealed significant shape differences between female and male guinea pig skulls (Rsq = 0.129; F (1,28) = 4.14; Z = 3.63; *p* = 0.001). Sex accounted for approximately 12.9% of the total variation in shape. The mean shapes of females and males differed with a Procrustes distance of 0.0261, indicating a clear morphological separation between the groups.

### 3.2. Shape Variation & Sex-Related Components (PC1–PC3)

**Figure 3:**
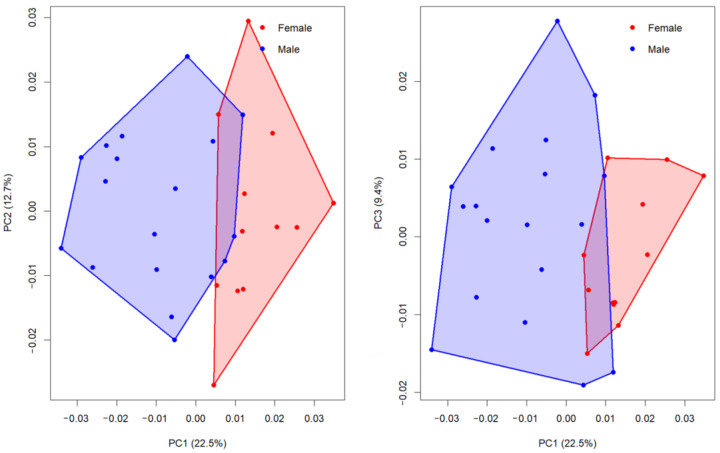
Scatterplots of PC1–PC2 (**left**) and PC1–PC3 (**right**) with convex hulls illustrating separation between females (red) and males (blue).

The first five principal components (PC1–PC5) explained 22.5%, 12.7%, 9.4%, 8.2% and 7.4% of the total shape variance, respectively, together accounting for 60.2% of the overall variation in cranial shape (Figure 3). This indicates that although variation is distributed across multiple axes, a substantial portion of the sex-related signal is concentrated in the first component.

Comparisons of group means revealed that only PC1 showed a statistically significant difference between females and males. Welch’s two-sample *t*-test confirmed a strong sexual dimorphism on PC1 (t = 5.77, df = 27.98, *p* < 0.0001, BH-adjusted *p* = 1.0 × 10^−5^). Female skulls displayed consistently higher PC1 scores (mean = 0.015) compared to males (mean = −0.010), with a mean difference of 0.024 (95% CI: 0.016–0.033). The corresponding effect size was very large (Cohen’s d = 1.97), highlighting a robust divergence in shape along this axis. In contrast, no significant group differences were observed for PC2 (*p* = 0.776, d = −0.11) or PC3 (*p* = 0.268, d = −0.39), both of which primarily reflected individual-level variation rather than sex-specific patterns.

To assess how well cranial shape discriminates between sexes, we performed LDA on the first three principal components of shape. Using leave-one-out cross-validation, the LDA correctly classified 25 of 30 individuals, corresponding to an overall accuracy of 83.3% (95% CI: 65.3–94.4%). The cross-validated confusion pattern was asymmetric between sexes: 11 of 12 females (91.7%) and 14 of 18 males (77.8%) were correctly assigned to their true sex. A permutation-based LOOCV test, in which sex labels were randomly permuted 999 times and the entire LDA + cross-validation procedure re-run on each permuted dataset, confirmed that this accuracy was significantly higher than expected by chance (permutation *p* = 0.001).

Taken together, these results indicate that PC1 is the primary dimension of shape variation associated with sexual dimorphism in guinea pig skulls, while PC2 and PC3 represent non-sexual morphological variation. The strong separation along PC1 suggests consistent differences in cranial morphology between females and males, which can be reliably captured in a geometric morphometric framework.

**Figure 4:**
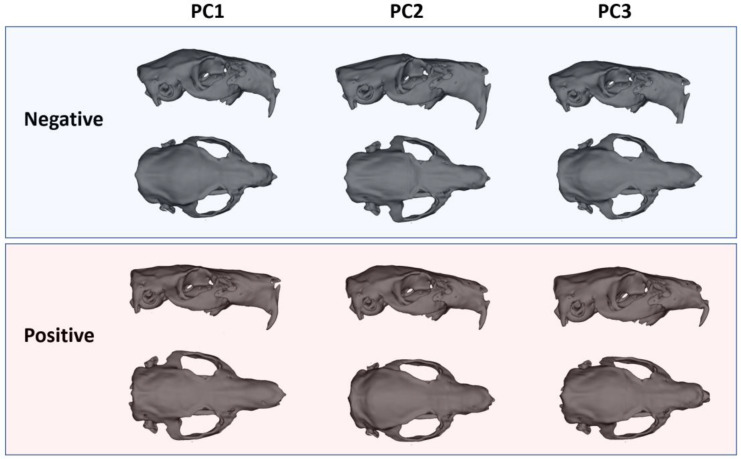
3D models represent cranial shape changes along the main axes of variation.

The principal component analyses demonstrated that the most prominent axis of variation distinguishing male and female guinea pig skulls was PC1, which accounted for 22.5% of the total shape variability. Along this axis, females consistently occupied positive values, while males clustered toward negative values, indicating a clear sex-related morphological signal (Figure 4). Female skulls were characterized by a more gracile and elongated form, with a narrower facial part, less lateral expansion of the zygomatic arches, and a slightly taller, more vaulted cranial roof. In contrast, male skulls displayed a markedly more robust configuration, with broader and more laterally projecting zygomatic arches and an expanded posterior cranial region.

In comparison, PC2 (12.7%) captured variation related mainly to the dorsal cranial profile and orbital positioning, while PC3 (9.4%) reflected changes in the relative breadth of the rostrum and neurocranial proportions (Figure 4). However, neither PC2 nor PC3 showed clear sex-specific clustering, indicating that these axes represent individual variation rather than consistent sexual dimorphism. The overall pattern, therefore, highlights that sexual shape dimorphism in guinea pigs is largely concentrated along PC1, reflecting differences in cranial robustness and functional morphology, whereas higher-order components capture subtle, non-sex-specific aspects of skull variation (Figure 4).

### 3.3. Allometry

The analyses revealed a clear allometric signal in the skulls of guinea pigs. Centroid size was strongly associated with cranial shape, explaining nearly 20% of the total variance (R^2^ = 0.197, F = 6.89, *p* = 0.001). Larger skulls exhibited consistent shape transformations relative to smaller ones. In contrast, body weight alone accounted for a relatively small proportion of cranial shape variation (Rsq = 0.104, F = 3.27, Z = 3.44, *p* = 0.001; based on the subset of individuals with recorded body weight). However, when both variables were entered into the same model, only centroid size remained significant, while body weight lost its explanatory power. A similar outcome was obtained in the log-transformed models, underscoring that cranial allometry is primarily driven by skull size rather than body weight.

To evaluate sexual allometry, we fitted a Procrustes model including log-transformed centroid size and sex. In this model, sex remained a significant predictor of cranial shape after accounting for size (F = 4.14, Z = 3.82, *p* = 0.001; R^2^_partial = 0.129), indicating that approximately 13% of cranial shape variation is associated with sex independently of overall skull size. Consistent with these findings, a linear regression of centroid size on body weight showed that body weight alone explained 52.9% of the variance in centroid size (R^2^ = 0.529, adjusted R^2^ = 0.512). When sex was added as an additional predictor, the explained variance increased to 64.7% (R^2^ = 0.647, adjusted R^2^ = 0.620), corresponding to an increase of ΔR^2^ = 0.118 (Δadjusted R^2^ = 0.108) relative to the single-predictor model.

**Figure 5:**
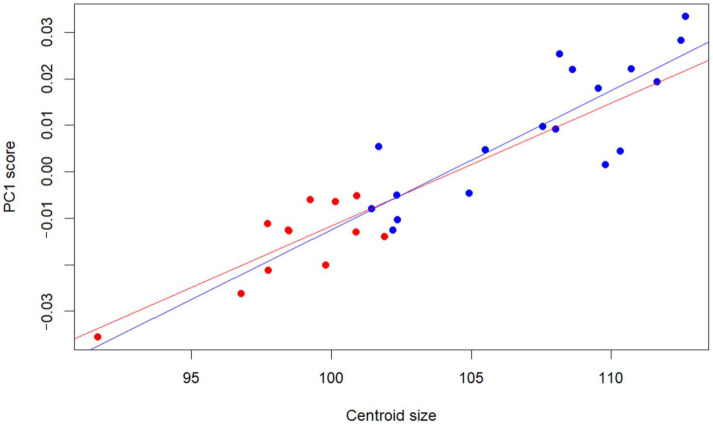
Relationship between PC1 scores and centroid size in male (blue) and female (red) guinea pigs.

The relationship between cranial shape and size was further explored by regressing PC1 scores on centroid size and sex (Figure 5). The model revealed a significant positive effect of centroid size on PC1 (Estimate = 0.0026, *p* = 0.0038), indicating that larger crania tend to occupy more positive positions along the main axis of shape variation. In contrast, neither the main effect of sex (*p* = 0.71) nor the interaction between centroid size and sex (*p* = 0.72) was significant, suggesting that males and females share a similar allometric trajectory along PC1. The model explained a large proportion of the variation in PC1 scores (R^2^ = 0.83), implying that differences in cranial shape captured by PC1 are strongly associated with overall size, rather than reflecting sex-specific differences in allometric slope.

Morphological inspection of the allometric vectors showed that as centroid size increased, the neurocranial region became proportionally more elongated, while the facial skeleton exhibited relative narrowing. In larger skulls, the orbits tended to be positioned slightly closer to the neurocranium, and the zygomatic arches extended more laterally, providing greater breadth in the cranial region. Conversely, smaller skulls retained a rounder cranial vault and a relatively shorter facial profile. These coordinated changes suggest that the allometric trajectory is associated with both functional and structural demands. Such size-related changes in the skull design are consistent with growth scaling of the masticatory apparatus and could also contribute to sex-biased differences in cranial robustness. In larger individuals, the lateral expansion of the zygomatic arches and the relatively elongated neurocranial region likely increase the mechanical advantage and cross-sectional area available for the jaw adductor muscles, thereby enhancing bite force and chewing efficiency. Such size-related changes in cranial design are consistent with growth scaling of the masticatory apparatus and could also contribute to sex-biased differences in cranial robustness, potentially reflecting a component of sexual selection on feeding performance or competitive behaviours.

Taken together, the results indicate that skull shape variation in guinea pigs is predominantly size-dependent, with body weight making a minimal additional contribution once overall cranial size is considered. This highlights a strong cranial allometric pattern, where morphological integration ensures proportional adjustments between neurocranial and facial regions as size increases.

## 4. Discussion

This study has completed all landmark-based statistical analyses, offering a comprehensive assessment of cranial shape variation in the domestic guinea pig. These analyses encompassed ANOVA for centroid size, Procrustes ANOVA for shape, allometry tests (multivariate regression of shape on size and body weight), and principal component analysis. ANOVA results revealed significant differences in centroid size between sexes, with males exhibiting larger skulls than females [31]. Moreover, Procrustes ANOVA confirmed a significant effect of sex on cranial shape, indicating that sexual dimorphism in guinea pigs extends beyond size to include distinct shape variation [25]. Regression analyses demonstrated that size significantly influences shape, revealing an allometric component to sexual dimorphism; however, residual shape differences between sexes persisted even after accounting for size, suggesting that this dimorphism is not solely a byproduct of scaling but reflects true shape divergence [32]. PCA further supported these findings by showing a tendency for males and females to occupy distinct regions of morphospace, largely explained by neurocranial breadth and rostral elongation.

The sexual dimorphism observed in guinea pigs parallels findings in other rodent species. For example, studies on brown rats (*Rattus norvegicus*) reported significant sexual dimorphism in both cranial shape and size, with males exhibiting larger skulls and region-specific differences [33]. Similarly, geometric morphometric analyses of rabbit skulls (Oryctolagus cuniculus) showed shape dimorphism even in the absence of significant size differences [26]. The magnitude of sexual shape dimorphism (R^2^ = 0.13) in C. porcellus is comparable to that in brown rats, where dimorphism is highly significant and enables 100% sex classification via discriminant function analysis [33], but larger than in rabbits, where the effect is often negligible [26]. These comparisons highlight that sexual dimorphism in cranial morphology is widespread among small mammals, although its magnitude and anatomical localization can vary [34]. Interestingly, some morphometric surveys using only linear measurements failed to detect sexual dimorphism, as in certain populations of R. norvegicus [35], highlighting the superiority of geometric morphometric techniques in capturing subtle shape differences [36]. Taken together, our results confirm that guinea pigs follow the male-biased dimorphism model observed in many rodents [31].

Allometry played a significant role in explaining skull variation in guinea pigs, as shape was strongly correlated with centroid size. Larger skulls displayed proportionally more robust zygomatic arches and expanded cranial vaults, whereas smaller skulls tended toward an elongated face. Such non-isometric scaling agrees with broader patterns in mammalian skull growth, where different regions exhibit differential growth rates [18]. Importantly, after statistically controlling for size, shape differences between sexes remained, suggesting structural or developmental divergence beyond simple scaling. These residual shape differences may arise from hormonal or developmental influences, such as varying levels of sex hormones affecting bone remodeling or differential timing in cranial ossification between males and females. This may reflect functional adaptations, such as differences in masticatory muscle attachment or feeding biomechanics between males and females. For instance, the robusticity in males corresponds to an increased surface area for the attachment of the masseter and temporalis muscles, suggesting a functional adaptation toward enhanced bite force. In larger individuals, the lateral expansion of the zygomatic arches and the relatively elongated neurocranial region likely increase the mechanical advantage and cross-sectional area available for the jaw adductor muscles, thereby enhancing bite force and chewing efficiency [37]. The persistence of sexual shape dimorphism after allometry correction reinforces the notion that cranial growth trajectories differ between sexes in guinea pigs, echoing findings in comparative morphometric studies across rodents [38].

These findings carry practical significance for veterinary anatomy and comparative biology. In clinical contexts, awareness of sex-specific cranial conformation can help veterinarians better interpret radiographic variations, such as differences in skull density or alignment, that may indicate sex-related anomalies. This awareness can also aid in managing dental or respiratory issues, which can be influenced by cranial architecture [39]. In zooarchaeological or forensic contexts, landmark-based cranial data can assist in identifying guinea pig remains and even facilitate forensic sexing of small mammals when pelvic bones are unavailable [40]. Moreover, the persistence of natural sexual dimorphism in a domesticated species, such as the guinea pig, underscores the stability of these traits despite artificial selection, suggesting that developmental or hormonal underpinnings are conserved within caviomorph rodents [31,41].

At first glance, our finding of pronounced male-biased differences in cranial size and shape appears to contrast with previous reports of relatively modest sexual dimorphism in guinea pig skull morphology. Several factors may help reconcile these patterns. First, most earlier studies relied on a limited set of external linear measurements or indices, which may underestimate more complex, multivariate shape differences that become evident when the entire cranium is analysed using 3D landmarks. Second, our sample consisted exclusively of skeletally mature adults (>1 year) from a single, well-nourished zoological colony, whereas some previous work combined different developmental stages or focused on specific laboratory lines. If sexual size and shape dimorphism is accentuated late in ontogeny or varies among domestic lines and husbandry regimes, our study may capture a portion of the variation that was not fully represented in earlier datasets. A minor limitation is that body weight data were unavailable for two individuals, so all body-weight–based allometric analyses were conducted on a slightly reduced sample. Finally, we examined both centroid size and global cranial shape, revealing coordinated changes in neurocranial and facial regions; such integrated patterns may not be detectable with traditional one- or two-dimensional metrics. Taken together, these considerations suggest that sexual dimorphism in guinea pig cranial morphology may be stronger than previously appreciated, at least in some domestic lines and at fully adult stages, and highlight the need for further comparative work across breeds, developmental stages, and measurement approaches. A limitation of the present study is the moderate sample size, which, although sufficient for statistical testing, may not capture the full range of population variation. Although the sample size (*n* = 30) was sufficient for detecting group differences, a larger dataset would further enhance generalizability. Additionally, only domestic guinea pigs were analyzed; comparisons with wild Cavia species could provide valuable evolutionary context.

## 5. Conclusions

The guinea pig skull represents a key anatomical structure with diagnostic relevance for both species identification and sex determination. This study is the first CT-based three-dimensional geometric morphometric analysis of the guinea pig skull, demonstrating that sexual dimorphism encompasses both size and shape, with males exhibiting larger and more morphologically distinct skulls than females. These findings are consistent with patterns observed in other rodents and underscore the utility of geometric morphometrics in detecting subtle yet biologically significant shape differences. Future research integrating finite-element modeling or developmental series could help link cranial shape variation to biomechanical performance, while broader comparisons across caviomorph rodents may elucidate the evolutionary pathways underlying sexual dimorphism of the skull.

## Figures and Tables

**Table 1:** Definitions for the 21 three-dimensional cranial landmarks digitized on guinea pig (*Cavia porcellus*) skulls.

1	Prosthion–the most rostral point of the upper incisor alveolar margin in the median plane.
2	Rhinion-the most rostral e most rostral point of the nasal bones in the median plane
3,4	The most lateral point of the incisive bone.
5,6	The most lateral point of the orbital margin of the frontal bone.
7	Premolare–the median point of the line joining the rostral points of the first premolar alveoli.
8	The most rostral point of the bony palate.
9	Midpoint of LM3 and LM4
10	Midpoint of LM5 and LM6
11,12	The most caudo-lateral point of the zygomatic arch.
13,14	Euryon–the most lateral point of the braincase.
15	The most dorsal point of the frontal bone.
16	External occipital protuberance.
17,18	The most lateral points of the nuchal crest.
19	Opisthion–dorsal mid-point of the foramen magnum on the occipital margin.
20	Basion–ventral mid-point of the foramen magnum on the basioccipital margin.
21	Staphylion–the most caudal point of the bony palate in the median plane.

## Data Availability

The data presented in this study are available upon request from the corresponding author (O.G.).

## Supplementary Materials

The following supporting information can be downloaded at: https://www.mdpi.com/article/10.3390/ani15233453/s1. Table S1: 3D landmark coordinates (x, y, z) and metadata (specimen ID, sex) for all 30 guinea pig skulls analysed in this study.
